# Comparison of the association between different ozone indicators and daily respiratory hospitalization in Guangzhou, China

**DOI:** 10.3389/fpubh.2023.1060714

**Published:** 2023-01-30

**Authors:** Geng Lin, Zhuoqing Wang, Xiangxue Zhang, Alfred Stein, Kamal Jyoti Maji, Changxiu Cheng, Frank Osei, Fiona Fan Yang

**Affiliations:** ^1^School of Geography and Planning, Sun Yat-sen University, Guangzhou, China; ^2^Department of Scientific Research and Discipline Development, The First Affiliated Hospital of Sun Yat-sen University, Guangzhou, China; ^3^State Key Laboratory of Earth Surface Processes and Resource Ecology, Beijing Normal University, Beijing, China; ^4^Faculty of Geo-Information Science and Earth Observation (ITC), University of Twente, Enschede, Netherlands; ^5^School of Civil and Environment Engineering, Georgia Institute of Technology, Atlanta, GA, United States; ^6^National Tibetan Plateau Data Center, Beijing, China

**Keywords:** O_3_ indicators, respiratory health, hospitalization visits, time-stratified case, air pollution

## Abstract

**Background:**

Epidemiological studies have widely proven the impact of ozone (O_3_) on respiratory mortality, while only a few studies compared the association between different O_3_ indicators and health.

**Methods:**

This study explores the relationship between daily respiratory hospitalization and multiple ozone indicators in Guangzhou, China, from 2014 to 2018. It uses a time-stratified case–crossover design. Sensitivities of different age and gender groups were analyzed for the whole year, the warm and the cold periods. We compared the results from the single-day lag model and the moving average lag model.

**Results:**

The results showed that the maximum daily 8 h average ozone concentration (MDA8 O_3_) had a significant effect on the daily respiratory hospitalization. This effect was stronger than for the maximum daily 1 h average ozone concentration (MDA1 O_3_). The results further showed that O_3_ was positively associated with daily respiratory hospitalization in the warm season, while there was a significantly negative association in the cold season. Specifically, in the warm season, O_3_ has the most significant effect at lag 4 day, with the odds ratio (OR) equal to 1.0096 [95% confidence intervals (CI): 1.0032, 1.0161]. Moreover, at the lag 5 day, the effect of O_3_ on the 15–60 age group was less than that on people older than 60 years, with the OR value of 1.0135 (95% CI: 1.0041, 1.0231) for the 60+ age group; women were more sensitive than men to O_3_ exposure, with an OR value equal to 1.0094 (95% CI: 0.9992, 1.0196) for the female group.

**Conclusion:**

These results show that different O_3_ indicators measure different impacts on respiratory hospitalization admission. Their comparative analysis provided a more comprehensive insight into exploring associations between O_3_ exposure and respiratory health.

## Introduction

Ozone (O_3_) is a secondary pollutant, forming through chemical reactions from precursors mainly including volatile organic compounds (VOCs) and nitrogen oxides (NOx). In recent years, O_3_ pollution has received increasing scientific attention due to the large number of environmental problems caused by rapid urbanization and industrial activities worldwide. Ozone (O_3_) exposure would trigger bronchial inflammation and respiratory tract oxidative stress, which further causes many serious health problems (1, 2) such as respiratory and lung-related diseases are very common (3–6).

Darrow et al. and Wise explored the relationship between O_3_ and respiratory health and provided their exposure-response coefficients but mainly in developed countries (7, 8). Developing countries, such as China and India, however, face severe O_3_ pollution and have a high population density (9, 10). Epidemiological studies about O_3_ concentration are still lacking, and therefore, there exists a lack of local exposure-response coefficients, resulting in large uncertainty in environmental health assessment. In addition, the relationship between O_3_ concentration and human health may vary across cities or regions because of differences in the nature and level of O_3_ pollution (10–12), and directly adopting the relationships established in developed countries to Chinese cities may result in large biases. It is, therefore, necessary to use local O_3_ concentration and health data to obtain local exposure-response coefficients.

With the improvement of the quality of China's air pollution monitoring data and increasing O_3_ concentrations (13), increasing attention can now be paid to the impact of O_3_ concentration on human health. Many epidemiological publications have confirmed that short-term O_3_ exposure is related to human health in China (4, 14, 15). However, few studies investigated how well the different O_3_ indicators (MDA8 O_3_ and MDA1 O_3_) measure the effects on human health. In fact, different O_3_ indicators have varying associations with human health (5, 16, 17). For example, Li et al. (16) used different O_3_ indicators to explore the impact of short-term O_3_ exposure on all-cause mortality in Guangzhou. Their results showed that MDA8 O_3_ was closely related to all-cause mortality, which was the key to study the impact of environmental O_3_ exposure on health (16). Yang et al. (17) examined the effect of three O_3_ indicators (MDA8 O_3_, MDA1 O_3_, and 24 h average O_3_) on daily mortality in Suzhou. They found that MDA8 O_3_ and MDA1 O_3_ were strongly associated with increased mortality than the 24 h average O_3_ (17). A cohort study by Abbey and Burchette investigated the impact of different O_3_ indicators on respiratory disease, and they found that MDA8 O_3_ provided the strongest impact on human health (18). As O_3_ concentration shows large diurnal and seasonal variation related to the variability in the release of O_3_ precursors, O_3_ indicators may be affected by spatial factors, such as region, urbanization, and population density, and temporal factors, such as season and weather. Therefore, in studies relating O_3_ to health, it is important to know how the O_3_ concentration is obtained. If the differences among O_3_ indicators are not considered appropriately, then this may lead to misleading health risk conclusions (16).

Previous studies examined the effects of short-term O_3_ exposure on daily all-cause, cardiovascular, and respiratory mortality (19–21), while a few studies addressed the problem that various O_3_ indicators were used to examine the relationship between different O_3_ indicators exposure and respiratory hospitalization.

To address this research issue, this study aimed (1) to conduct a time-stratified case–crossover model to explore the short-term effect of two O_3_ indicators (MDA8 O_3_ and MDA1 O_3_) on daily respiratory hospitalization in a single city; (2) to investigate whether the two O_3_ indicators show different relationships with daily respiratory hospitalization; and (3) to examine the associations between the two O_3_ indicators and daily respiratory hospitalization for different age, gender, and season groups. Then, the results of the single-day lag model and the moving average lag model were compared. As the study area, we selected the city of Guangzhou, China, where an excellent dataset was available.

## Materials and methods

### Data collection

**Figure 1:**
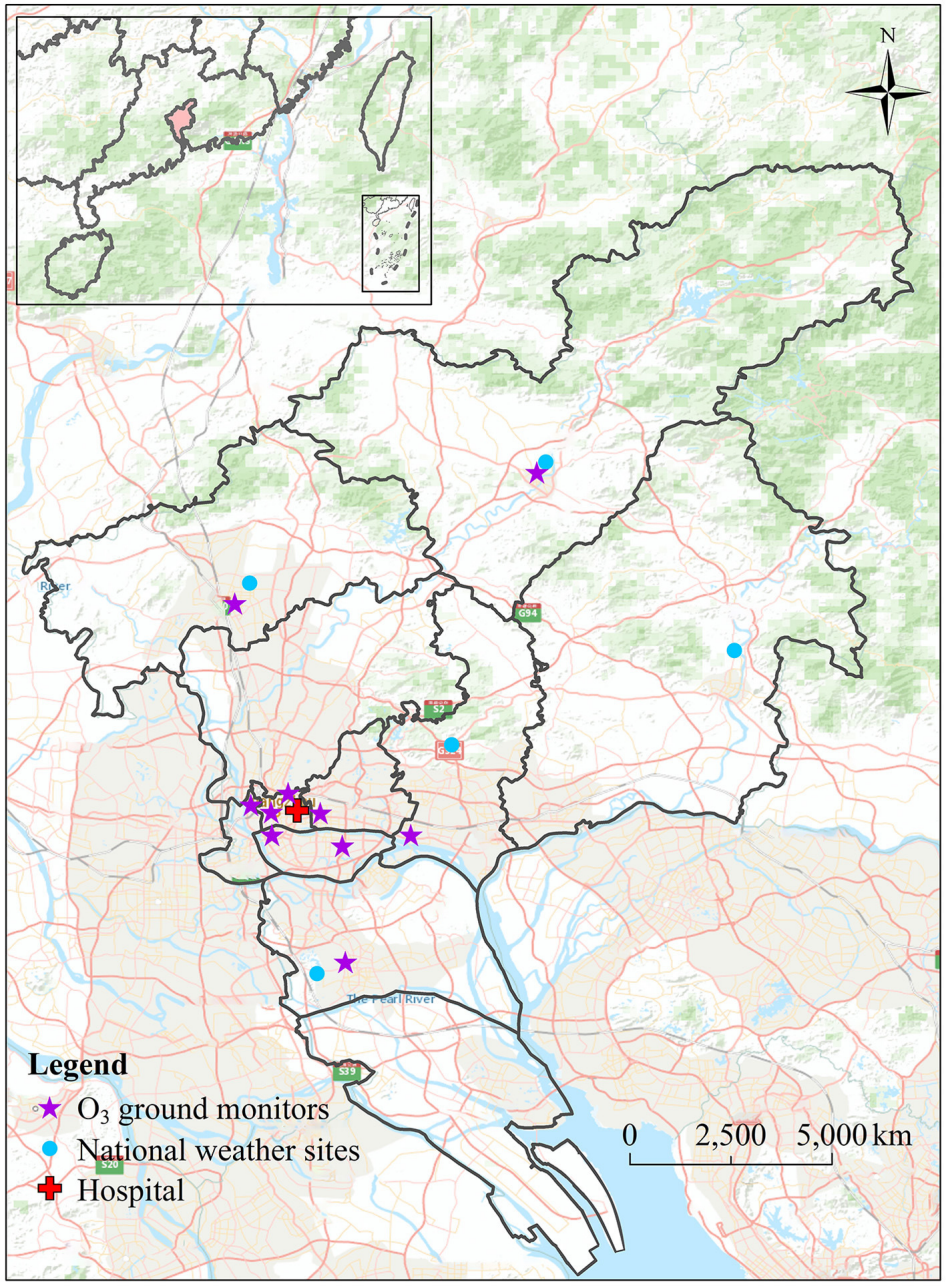
The spatial distribution of air quality monitoring stations, weather sites, and targeted hospital in Guangzhou.

Guangzhou, located in Southern China, is a metropolis with a high population density and high O_3_ concentration. It belongs to the typical subtropical humid monsoon climate, and its annual average temperature is 22°C and relative humidity is 68%. Due to the rapid economic development and increased energy consumption in the past few decades, Guangzhou has suffered from severe air pollution. Moreover, Guangzhou is a typical Chinese megacity, representing a city with urgent public health problems caused by air pollution. Therefore, Guangzhou is a unique city to evaluate the health effects of O_3_ concentration. We collected data on daily respiratory hospitalization from 1 January 2014 to 31 August 2018 from the First Affiliated Hospital of Sun Yat-sen University, which is located in Yuexiu District, central district of Guangzhou (Figure 1). This hospital is surrounded by universities, a large community of residents. Therefore, the daily respiratory hospitalization in this hospital can reflect the changes in the respiratory health status of residents in Guangzhou. The information on respiratory data contains the date of hospitalization, gender, age, diagnosis from the 10th International Classification of Diseases (ICD-10), and residential address. In this study, respiratory diseases (ICD-10: J00-J99) include upper and lower respiratory tract diseases. Since 89.4% of people in this dataset were above the age of 15, therefore, in this study, hospitalization visits were divided into two groups: 15–60 years (15 ≤ age < 60 years old) and 60+ years (≥60 years old). The screening was performed according to the patient's residential address, and the inpatients in this study were all local residents of Guangzhou.

Data on air pollutants include the two O_3_ indicators (MDA8 O_3_ and MDA1 O_3_), NO_2_, and PM_2.5_ concentration in Guangzhou from 1 January 2014 to 31 August 2018, which were collected from the air quality monitoring sites in Guangzhou. The data are published by the China National Environmental Monitoring Centre (http://quotsoft.net/air/). Because the hospitalized patients come from different districts of Guangzhou, the average value of 10 pollutant monitor sites was used. Data on PM_2.5_ and NO_2_ were used to test the sensitivity of the relationship between different O_3_ indicators and respiratory hospitalization in the multi-pollutant model.

Meteorological factors were obtained from the China Meteorological Data Sharing Service System (http://data.cma.gov.cn/). Daily average temperature and relative humidity were included to adjust for meteorological effects on respiratory hospitalization. All data are divided into two periods: warm and cold periods, based on the average temperature of each month, that is, months with an average temperature above 20°C are classified as warm period (May to October) and those below 20°C as cold period (November to April).

### Statistical analysis

We applied a time-stratified case–crossover (TSCC) design in this study. The case–crossover design combines the advantages of case–control and cross-sectional studies and can be seen as an extension of the traditional case–control design (22). It uses the case itself as the control to avoid the bias caused by the selection of the control group and some uncontrollable factors between cases (such as age, intelligence, and job). Compared with traditional case–control studies, case–crossover designs have advantages in controlling for time-invariant confounders of individual characteristics, since each individual is under his or her own control (23). The TSCC design has been widely used to examine the impact of air pollution or extreme weather conditions on health outcomes such as morbidity and mortality (24–26).

We used a TSCC design to examine the relationship between two different O_3_ indicators and daily respiratory hospitalization. Of these, all cases served as their own controls. Odds ratios (ORs) and their 95% confidence intervals (CIs) between short-term O_3_ exposure and daily respiratory hospitalization were calculated using a conditional logistic regression model that was conducted with a Cox proportional hazards regression model. If the *p*-values were < 0.05, the results were considered statistically significant. The formula was as follows:


(1) $$\begin{array}{l} \log\left(h\left(t,X\right)\right)\;=\;\log\left(h_{0}\left(t\right)\right)+\beta_{1}C_{t}+\beta_{2}AT+\beta_{3}RH \end{array}$$

where *log* (*h* (*t, X*)) is the risk function of exposure to the independent variable *X* (*X* includes pollutants and meteorological factors) on day *t* (*t* is the date of hospital admission), *log*(*h*_0_(*t*)) is the baseline risk function, *C*_*t*_ is the daily O_3_ concentrations, *AT* is the average temperature, and *RH* is the relative humidity, with coefficients β_1_, β_2_, and β_3_. The results are presented as changes in percentage and their 95% CI of hospital admissions by a rise of per interquartile range (IQR) in O_3_ concentration. We choose the same day of the week 1 month before the patient's admission as a control. For instance, if patient visits on a Tuesday in June 2016, all Tuesdays a month ago are control days. According to this design, each case has 3–4 control days (27).

To capture the delayed (or “lag”) effects of O_3_ on respiratory hospitalization, we investigated the delayed associations of O_3_ exposure on hospitalization visits in Guangzhou. We both used a single-day lag model (lag0 to lag5), moving the average lag model to explore their cumulative effects (lag01 to lag05). Notably, the O_3_ concentration of lag0 refers to the concentration of O_3_ on the current day, and lag1 refers to the O_3_ concentration of the previous day. The O_3_ concentration of lag01 was calculated by the 2-day (the current day and the previous day) average, and similarly, the O_3_ concentration of lag05 was calculated by the average O_3_ concentration on the current day and the 5 days ago.

We performed a series of subgroup analyses stratified by age (15–60 years old and 60+ years old) and gender (male and female) to identify potentially susceptible subgroups. These age stratifications refer to the division from the previously published studies (4, 28). Moreover, a seasonal analysis of O_3_-related effects was also performed by dividing the entire study period into warm (May–October) and cold (November–April) periods. In these subgroup analyses, the effects of different O_3_ indicators on daily respiratory hospitalization in Guangzhou were examined separately.

### Sensitivity analysis

To test the stability of these results, we performed the following sensitivity analyses: (1) Multi-pollutants analyses were performed by including the other two serious pollutants, NO_2_ and PM_2.5_; (2) change the lag days of meteorological factors (temperature and relative humidity) from 0 to 3, which were used to check whether the results are sensitive to changes in meteorological factors. All the aforementioned analyses were performed using the “survival” package in R software (version 3.6.3).

## Results

### Descriptive analysis

Table 1 describes the basic characteristics of daily respiratory hospitalization data, two different O_3_ indicators, two other pollutants (PM_2.5_ and NO_2_), and meteorological factors (average temperature and relative humidity). The cumulative number of respiratory hospitalization visits was equal to 5,229 cases from 1 January 2014 to 31 August 2018, and the average number of hospitalization visits per day was 6. Approximately 62.6% of patients were male and 37.4% were female. The number of hospitalization visits for respiratory diseases in the age group of 15–60 (44.1%) was slightly lower than that in the 60+ age group (45.2%) (Table 1).

**Table 1:** Descriptive statistics of different O_3_ indicators, meteorological factors and hospitalization for respiratory diseases in Guangzhou.

**Variable**	**Min**	**25%**	**50%**	**Mean**	**SD**	**75%**	**IQR**	**Max**
MDA8 O_3_ (μg/m^3^)	4	51	86	91	51	122	71	271
MDA1 O_3_ (μg/m^3^)	4.3	57	100	105	59	144	87	311
PM_2.5_ (μg/m^3^)	4	23	33	38	22	49	26	157
NO_2_ (μg/m^3^)	14	33	42	46	18	55	22	148
Temperature (°C)	3.3	17.1	23.9	22.2	6.3	27.7	10.6	31.7
Relative humidity (%)	28	73	80	79	10	86	13	97
RS hospital admission (number)	0	2	6	6	5	9	7	23

RS, represents respiratory.

Detailed information on air pollutants and meteorological factors from 2014 to 2018 in Guangzhou is listed in Table 1. From 2014 to 2018, the daily concentration of MDA8 O_3_ and MDA1 O_3_ ranged from 4.0 to 271.0 μg m^−3^ and 4.3 to 311.1 μg m^−3^, respectively. The annual average value of PM_2.5_ was 38.2 μg m^−3^, which was 9.14% higher than the Grade II Annual Standard (35 μg m^−3^) of the Chinese Ambient Air Quality Standards (CAAQS) and 2.8 times higher than the annual average value (10 μg m^−3^) reported by the World Health Organization. The daily concentration of NO_2_ ranges from 14 to 148 μg m^−3^, with an annual average of 46.1 μg m^−3^. From 2014 to 2018, there are 907 days that NO_2_ exceeded the Grade II Annual Standard CAAQS (40 μg m^−3^). Notably, PM_2.5_ and NO_2_ concentrations were higher in the cold season than that in the warm season, but O_3_, on the contrary, was higher in the warm season than that in the cold season. In addition, the average daily temperature was included as a confounder in the model, ranging from 3.3 to 31.7°C (the annual average is 22.2°C). Daily relative humidity ranges from 28 to 97% (the annual average is 78.7%). Specifically, the statistical characteristics of four pollutants, MDA8 O_3_, MDA1 O_3_, PM_2.5_, and NO_2_, and two meteorological factors, average temperature and relative humidity, are shown in Table 1.

### Comparison of all different age and gender groups

**Figure 2:**
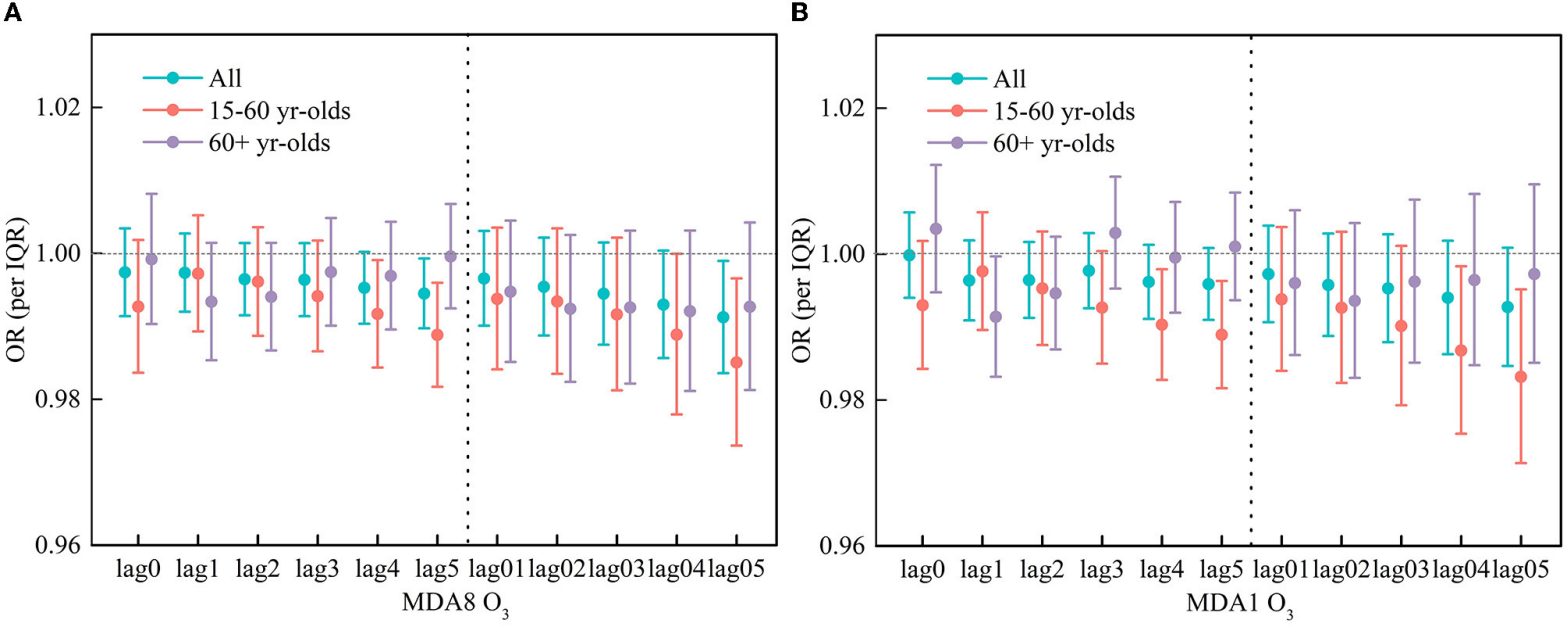
OR of respiratory hospitalization visits in different age group for per IQR increase in MDA8 O3 **(A)** and MDA1 O3 **(B)**.

Figure 2 shows the relationships between two O_3_ indicators and respiratory hospitalization visits in a single-pollutant model. At lag 5 day, MDA8 O_3_ has a significant and negative impact on the risk of respiratory hospitalization visits, with the OR value of 0.9945 (95% CI: 0.9897, 0.9993). As for MDA1 O_3_, there is no significant impact for all people.

The respiratory risk in different age groups was further examined by applying the same model to identify whether there are differences in different stratifications. As shown in Figure 2A, MDA8 O_3_ was inversely associated with respiratory hospitalization visits in Guangzhou. At the lag of 4 and 5 days, MDA8 O_3_ had a significant impact on respiratory hospitalization in the 15–60 age group, and the OR value was 0.9917 (95% CI: 0.9843, 0.9991) and 0.9888 (95% CI: 0.9817, 0.9960), respectively. As for lag 04 and lag 05, there also have significant OR values, and they are 0.9889 (95% CI: 0.9779, 0.9999) and 0.9850 (95% CI: 0.9736, 0.9966), respectively. Meanwhile, no significant correlation was observed for the effect of MDA8 O_3_ on the 60+ age group.

Figure 2B shows the association between MDA1 O_3_ and respiratory hospitalization visits in different age groups in a single-pollutant model. At the lag4 and lag5 days, there was a significant effect of MDA1 O_3_ on the 15–60 years old, with values of 0.9903 (95% CI: 0.9828, 0.9979) and 0.9889 (95% CI: 0.9816, 0.9963). As for the 60+ age group, at the lag 1 day, the OR value is 0.9914 (95% CI: 0.9832, 0.9997).

**Figure 3:**
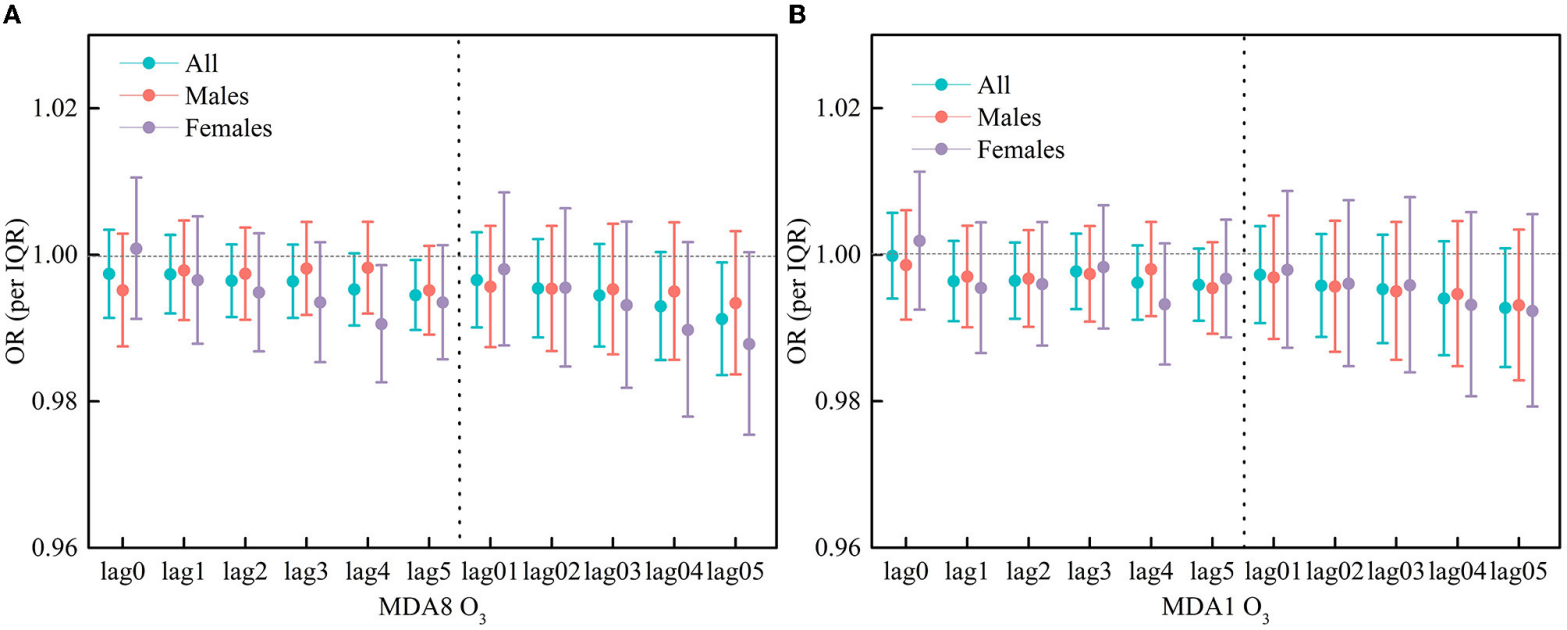
OR of respiratory hospitalization visits in different gender group for per IQR increase in MDA8 O3 **(A)** and MDA1 O3 **(B)**.

Figure 3 shows OR estimates for subgroups stratified by gender. No obvious differences were identified. Specifically, in almost all gender subgroups, there was no significant association between MDA8O_3_/MDA1O_3_ and respiratory hospitalization visits except for MDA8 O_3_ in the OR at the lag4 day for the female group, and its significant OR value is 0.9905 (95% CI: 0.9826, 0.9986).

In our analysis, the effect of O_3_ on respiratory hospitalization was statistically significant for some lag days. It can be seen that there is a different lagged effect of O_3_ on daily respiratory hospitalization in Guangzhou. In the different lag days, the effect of O_3_ on the respiratory hospitalization visits was different. Taking MDA8 O_3_ as an example for the 15–60 age group, the single-day lag effect at the lag4 has the greatest impact, that is, when the O_3_ concentration delay at 4 days, it has the greatest impact on the number of respiratory hospitalization visits. No significant effect, however, was observed in the 60+ age group. For women, the single-day lag effect was greatest at lag4; for men, no significant effect was observed. Moreover, compared with the single-day lag effect, the OR value of the moving average lag model is not significant, which indicates that for the whole year, the cumulative effect of O_3_ on respiratory hospitalization visits is also not significant.

### The influence of seasonal effects

O_3_ concentration has obvious seasonal variation. Due to the strong sunlight in summer and the strong photochemical reaction at high temperature, the phenomenon of high O_3_ concentration in summer and low O_3_ concentration in winter is formed. Therefore, we analyzed the effect of O_3_ during the warm period (May–October) and the cold period (November–April). Notable differences were identified during different periods. Specifically, in the warm period, there are positive and significant associations between MDA8 O_3_/MDA1 O_3_ and respiratory hospitalization visits; conversely, there is a negative association in the cold period.

**Figure 4:**
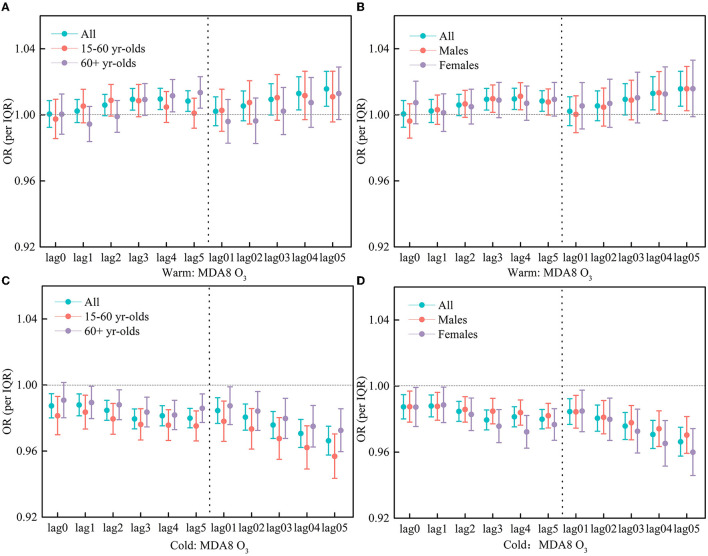
**(A–D)** OR of respiratory hospitalization visits for an increase of per IQR in MDA8 O3 during the warm periods (May to October) and the cold periods (November to April).

**Figure 5:**
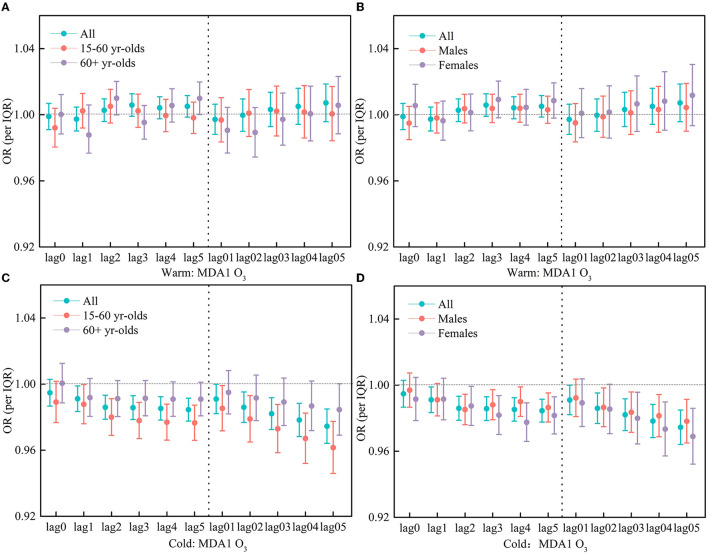
**(A–D)** OR of respiratory hospitalization visits for an increase of per IQR in MDA1 O3 during the warm periods (May to October) and the cold periods (November to April).

In the warm season, as for MDA8 O_3_ and all people, at the lag of 3–5 days, there have significant and positive associations, with the OR values being 1.0094 (95% CI: 1.0029, 1.0160), 1.0096 (95% CI: 1.0032, 1.0161), and 1.0084 (95% CI: 1.0021, 1.0146), respectively. As for the moving average lag model, there has a greater association at the lag 04 and lag05 days, and their OR values were 1.0130 (95% CI: 1.0030, 1.0231) and 1.01573 (95% CI: 1.0052, 1.0263), respectively (Figure 4). As for MDA1 O_3_ and all people, at the lag of 3–5 days, there is also a positive association but not statistically significant (Figure 5).

While in the cold season, MDA8 O_3_/MDA1 O_3_ was significantly and negatively correlated with respiratory hospitalization visits, both peaked at the lag05 day, with the OR being 0.9663 (95% CI: 0.9576, 0.9751) and 0.9745 (95% CI: 0.9641, 0.9849), respectively (Figures 4, 5).

The associations between short-term O_3_ exposure and respiratory hospitalization in different age groups were further examined in the warm period. At the lag 4 and 5 days, MDA8 O_3_ had a significant and positive impact on the respiratory hospitalization for the 60+ age group, with the OR being 1.0116 (95% CI: 1.0018, 1.0214) and 1.0135 (95% CI: 1.0041, 1.0231), respectively. For MDA1 O_3_, at the lag5 day, there is also a significant association, with the OR being 1.0099 (95% CI: 1.0001, 1.0198). For MDA8 O_3_/MDA1 O_3_, there is no significant association for the 15–60 age group of people (Figures 4, 5).

In different gender groups, the associations between O_3_ exposure and respiratory hospitalization were also examined. At the lag3, lag4, lag04, and lag05 days, MDA8 O_3_ had a significant and positive impact on respiratory hospitalization for the male group, with the OR values being 1.0097 (95%CI: 1.0015, 1.0181), 1.0112 (95%CI: 1.0030, 1.0194), 1.0133 (95%CI: 1.0006, 1.0262), and 1.0158 (95% CI: 1.0024, 1.0293), respectively. There is also a positive association for the female group but not statistically significant. Meanwhile, no significant association was observed for the effect of MDA1 O_3_ on different gender groups (Figures 4, 5).

### Sensitivity analysis

Sensitivity analyses demonstrated the robustness of our main findings. In addition to the single-pollutant model for O_3_, we also tested a multi-pollutant model, including PM_2.5_ and NO_2_. After adding PM_2.5_ and NO_2_ as risk factors, the OR value of the single-pollutant model for O_3_ did not change much. The OR values changed to −0.26, 0.20, −0.26, and 0.18%, respectively. The OR values of MDA1O_3_ changed to −0.01, 0.32, 0.01, and 0.26%, respectively.

In addition, after adjusting for different lagged days of meteorological factors, the results of the single-pollutant model for MDA8 O_3_/MDA1 O_3_ did not change much, and their values for MDA8 O_3_ were −0.319, −0.065, 0.045, and 0.147%, respectively. The OR value of MDA1O_3_ changed values were −0.016, 0.147, 0.223, and 0.308%, respectively. These results were not materially affected, suggesting that these results of this study are relatively stable.

## Discussion

Ozone had a negative impact on human health (29). To our knowledge, this is one of the few studies in China reporting the effects of two different O_3_ indicators on daily respiratory hospitalization visits. In this study, a time-stratified case–crossover design was used to evaluate the short-term effects of MDA8 O_3_/MDA1 O_3_ on daily respiratory hospitalization. The results showed that in the warm period, MDA8 O_3_ indicator appeared to be strongly associated with respiratory hospitalization visits risk than the MDA1 O_3_ indicator. The health effects of O_3_ on respiratory hospitalization visits are stronger in the 60+ age group than that in the 15–60 age group, and the female group is slightly more sensitive than the male group. Moreover, the association of both MDA8 O_3_ and MDA1 O_3_ and daily respiratory hospitalization visits was significant and positive in the warm period and negative in the cold period. Our findings help to understand the short-term health impact associated with different O_3_ indicators in Guangzhou, China, and present differences in the effects of O_3_ on different age and gender groups.

A comparative analysis of the two O_3_ indicators provided a comprehensive perspective to explore the relationships between O_3_ and respiratory health. From the year 2000 onward, various studies on respiratory mortality as measured globally by different indicators are reviewed in Table 2. These provide a comparative analysis, and we note large differences in the results. In the time-series analysis presented in this study, we observed different estimates for the MDA8 O_3_ and MDA1 O_3_ indicators. These were significant and negative and were associated with daily respiratory hospitalization visits at the lag 5 day, while MDA8 O_3_ had stronger associations than MDA1 O_3_. These results were similar to those obtained in previous studies. For instance, using a Poisson generalized linear model, Darrow et al. examined the association between daily respiratory emergency department visits and various O_3_ indicators (7). Their study showed that MDA8 O_3_ and MDA1 O_3_ were positively related to daily respiratory emergency department visits and that MDA8 O_3_ had a stronger association than MDA1 O_3_. Meanwhile, their OR values for MDA8 O3 and MDA1 O3 were greater than that in this study (Table 2). Moreover, Sun et al. conducted an epidemiological study on 34 counties in China exploring the associations between short-term exposure to different O_3_ indicators and respiratory mortality using three types of O_3_ indicators (MDA8 O_3_, MDA1 O_3_, and daily average) from 2013 to 2015 (32). Their results showed that the association between MDA8 O_3_ and respiratory mortality was stronger than that between MDA1 O_3_ and respiratory mortality, which is consistent with the results of this study. Notably, their OR value for MDA8 O_3_ was similar to that in our study, while the OR value was larger than that in our study. The potential reason for differences among the OR values of other studies and our study is that each city/region has different O_3_ levels and characteristics, as well as different population exposure patterns. Therefore, short-term exposure to different levels of O_3_ would have different health effects. Meanwhile, a possible explanation for MDA8 O_3_ had a greater impact is that the MDA8 O_3_ may be the most relevant indicator for individual exposure levels, as many people pour into the city during the day and move out at night. Moreover, high O_3_ exposure in the city during the daytime and 8-h maximum period may have higher health impacts than commute time and nighttime (16). Therefore, it has been suggested that health effects are related to short-term exposure to slightly higher O_3_ concentrations, such as MDA8 O_3_, rather than peak concentrations, such as MDA1 O_3_ (16).

**Table 2:** Various studies on respiratory mortality attributed to different O_3_ indicators after the year 2000 worldwide.

**References**	**Study year**	**Study area**	**Model**	**O_3_ indicator**	**Health endpoints**	**Estimates (β) (%)**
Lin et al. (30)	2000–2009	Taiwan, China	DLNM	MDA8 O_3_	D_RS_	5.0 (4.0, 5.01)
				MDA1 O_3_	D_RS_	2.0 (1.0, 2.01)
				24 h average	D_RS_	3.0 (2.0, 3.01)
Shi et al. (31)	2013–2018	128 counties, China	GLM	MDA8 O_3_	M_RS_	0.50 (0.31, 0.68)
				MDA1 O_3_	M_RS_	0.41 (0.25, 0.57)
				24 h average	M_RS_	0.89 (0.58, 1.19)
Sun et al. (32)	2013–2015	34 counties, China	DLNM	MDA8 O_3_	M_RS_	0.22 (−0.28, 0.72)
				MDA1 O_3_	M_RS_	0.11 (−0.22, 0.44)
				24 h average	M_RS_	0.57 (−0.09, 1.23)
Yang et al. (17)	2006–2008	Suzhou, China	GAM	MDA8 O_3_	M_RS_	−0.31 (−1.19, 0.53)
				MDA1 O_3_	M_RS_	−0.57 (−1.33, 0.16)
				24 h average	M_RS_	−0.70 (−2.18, 0.74)
Byers et al. (33)	2007–2011	USA	GAM	MDA8 O_3_	EDVA	1.37 (−0.10, 2.88)
				MDA1 O_3_	EDVA	1.41 (0.60, 2.78)
Darrow et al. (7)	1993–2004	Atlanta, USA	GLM	MDA8 O_3_	REDV	1.7 (1.0, 2.4)
				MDA1 O_3_	REDV	1.4 (0.8, 2.0)
				24 h average	REDV	1.1 (−0.1, 2.4)
Kazemiparkouhi et al. (34)	2000–2008	USA	LLRM	MDA8 O_3_	M_RS_	1.64 (1.49, 1.83)
				MDA1 O_3_	M_RS_	1.49 (1.34, 1.69)
				24 h average	M_RS_	1.04 (0.80, 1.29)
Mar et al. (35)	1998–2002	USA	GAM	MDA8 O_3_	EDVA	3.92 (0.99, 5.36)
				MDA1 O_3_	EDVA	2.96 (0.50, 5.36)
Gryparis et al. (36)	1990–1996	23 cities, Europe	GAM	MDA8 O_3_	M_RS_	1.13 (0.74, 1.51)
				MDA1 O_3_	M_RS_	1.13 (0.62, 1.48)
Moshammer et al. (37)	1991–2009	Vienna, Austria	GAM	MDA8 O_3_	M_RS_	1.29 (0.43, 2.15)
				MDA1 O_3_	M_RS_	1.29 (0.55, 2.04)
				24 h average	M_RS_	1.07 (0.01, 2.15)
Nhung et al. (38)	2007–2014	Vietnam	GAM	MDA8 O_3_	D_RS_	0.28 (−0.32, 0.87)
				24 h average	D_RS_	0.20 (−0.29, 0.68)

D_RS_, respiratory diseases; M_RS_, respiratory mortality; EDVA, emergency department (ED) visits for asthma; REDV, Respiratory emergency department visits; LLRM, log-linear regression model.

Strong sunlight in summer and high photochemical production at high temperature result in high O_3_ concentration in summer and low O_3_ concentration in winter (13, 39). Therefore, we analyzed the effect of the two O_3_ indicators on the daily respiratory hospitalization visits in Guangzhou, China, during the warm period (May–October) and the cold period (November–April). The positive associations in the warm period and the negative ones in the cold period were identified, which were consistent with previous studies. In both periods, the sensitivity to O_3_ concentration was also studied in men and women aged 15–60 and older than 60 years, respectively. We found that during the warm period, MDA8 O_3_ was significantly positively correlated with the daily respiratory hospitalization visits at lag3–lag5, with a higher risk occurring in the 60+ age group. The relationship between MDA1 O_3_ and daily respiratory hospitalization visits was almost insignificant. During the cold period, the two O_3_ indicators were significantly and negatively associated with respiratory hospitalization visits, with a higher risk in the 60+ age group than that in the 15–60 age group. These results are consistent with studies from other time-series studies (40).

For example, Wang et al. showed that O_3_ was positively related to respiratory outpatient visits in the warm period but negatively associated with that in the cold period (4), while Malig et al. showed that O_3_ exposure was significantly and positively related to respiratory emergency department visits, and there has a slightly larger association in the warm period (41). The possible reason is that people tend to go outdoors/open windows in the warm period, so people are easily exposed to such a high O_3_ environment at that case, which will have larger effects on human health. Li et al. showed that the association between O_3_ and daily mortality in Guangzhou seems to be more prominent in the cold period than in the warm period (16), while Yang et al. also demonstrated that O_3_ had a significant effect on human health as in the cold period, and the relationship between O_3_ and daily mortality seemed to be more evident than that in the warm period (17). Other studies showed that there is no significant relationship between O_3_ and human health during the warm period (42, 43). This difference with the results in this study is possibly caused by different interactions of O_3_ exposure and season at different locations. Other factors such as exposure patterns and levels of local residents, air conditioners usage (44), and ventilation rates between indoor and outdoor may influence the season to modify the relationship between daily O_3_ concentration and respiratory hospitalization visits.

Lag effects of O_3_ on the daily respiratory hospitalization visits may exist in Guangzhou, China. Compared with the single-day lagged model, O_3_ showed similar impacts in the results of the moving average lag model for different age and gender subgroups. Especially in the warm period, the OR of the single-day lag model is slightly lower than that of the moving average lag model, indicating that O_3_ also had a cumulative effect on respiratory hospitalization visits, which were similar to the previous studies (4, 45). The reason for the lag effects may be that O_3_ produces an acute inflammatory response in the lungs. Studies have demonstrated that this inflammatory response is caused by repeated exposure over several days (46, 47). Notably, inflammation may play a key role in the increased O_3_-related mortality and morbidity (48).

There are some limitations to this study. First, data on the ambient O_3_ were averaged using fixed monitoring sites rather than individual measurements, which can result in underestimating the health effects of O_3_. Second, our data on respiratory hospitalization visits came from a large comprehensive and famous hospital, rather than from all hospitals in Guangzhou, which may lead to the underestimation of O_3_ effects on respiratory diseases, reflected in relatively small OR values. Therefore, caution should be paid when generalizing the results to other regions. Third, although we adjusted the confounders such as meteorological conditions (average temperature and relative humidity), there is still the possibility that some unmeasured confounders could have influenced the results. Finally, we do not have any information about patients' smoking behavior, which is also responsible for respiratory problems.

## Conclusion

This study analyzed the relationship between daily respiratory hospitalization visits and two common O_3_ indicators in Guangzhou, China. It showed that the two O_3_ indicators were significantly and positively related to respiratory hospitalization visits in the warm period, and negatively in the cold period. In the warm period, women were more sensitive to O_3_ than men, and the 60+ age group was more sensitive than the 15–60 age group. Both the single-day lag model and the moving average lag model showed a significant effect on respiratory hospitalization visits. In the multi-pollutant model, adding one or all pollutants as a risk factor, the results for two different indicators are similar, indicating that the results are stable. These findings provide a comprehensive insight into the impact of different O_3_ indicators on human health in densely populated cities. They may serve as well as a reference for local governments to formulate air pollution measures to optimize emergency medical resources.

## Data availability statement

The original contributions presented in the study are included in the article/supplementary material, further inquiries can be directed to the corresponding authors.

## Author contributions

XZ and KM: conceptualization and methodology. GL, ZW, FY, and XZ: data curation, resources, and formal analysis. XZ: writing the original draft preparation. AS, CC, KM, and FO: supervision and writing, reviewing, and revising the manuscript. All authors contributed to the article and approved the submitted version.
